# Cases of Abnormally High Temperature

**Published:** 1880-07

**Authors:** 


### CASES OF ABNORMALLY HIGH TEMPERATURE.

A late number of the *British Medical Journal* contains a report by Dr. Donkin of eight cases of abnormally high temperature, all but one in females, and none proving fatal. Pain was a prominent symptom in all. An abbreviated statement is subjoined:

No. 1, 111.6°; convalescing from enteric fever.
No. 2, 108°; no organic lesions; ovarian pain.
No. 3, 115.8°; great abdominal pain and excitement.
No. 4, 111°; convalescing from enteric fever.
No. 5, 113°; enteric fever and double pneumonia.
No. 6, 112°; synovitis. This was the only male.
No. 7, 112°; painful stump, with necrosis.
No. 8, 117°; pyonephrosis.

